# Síndrome Landau-Kleffner y Síndrome de Punta Onda Continua Activada en Sueño: Comparación de Características Clínicas, Neurofisiológicas y Neuropsicológicas

**DOI:** 10.31083/RN33484

**Published:** 2025-08-26

**Authors:** Cristina Benítez-Provedo, Elena González-Alguacil, Marta García-Fernández, Borja Esteso Orduña, Nuria Lamagrande Casanova, Juan José García Peñas, Anna Duat Rodríguez, Víctor Soto-Insuga

**Affiliations:** ^1^Servicio de Neurología, Hospital Universitario Niño Jesús, 28009 Madrid, España; ^2^Servicio de Neurofisiología, Hospital Universitario Niño Jesús, 28009 Madrid, España. Facultad de Medicina, Universidad Francisco de Vitoria, Pozuelo de Alarcón, Madrid, España; ^3^Unidad de Neuropsicología Clínica, Servicio de Psiquiatría y Psicología Clínica de la Infancia y Adolescencia, Hospital Universitario Niño Jesús, 28009 Madrid, España

**Keywords:** encefalopatía epiléptica, Síndrome de Landau-Kleffner, punta-onda continua activada en sueño (SWAS, del inglés Spike Wave Activated in Sleep), afasia epiléptica adquirida, epileptic encephalopathy, Landau-Kleffner Syndrome, continuous spike-and-wave during sleep (SWAS), acquired epileptic aphasia

## Abstract

**Introducción:**

El Síndrome de Landau-Kleffner (SLK) es un subtipo de encefalopatía epiléptica con punta-onda activada durante el sueño (EE-SWAS) que se caracteriza por presentar una afasia adquirida asociada a la aparición de anomalías epileptiformes.

**Pacientes y Métodos:**

Estudio transversal-descriptivo en un hospital terciario con un grupo de niños con SLK y otro con EE-SWAS. El objetivo fue comparar los aspectos clínicos, neurofisiológicos y neuropsicológicas de ambos.

**Resultados:**

Se analizaron 7 pacientes con SLK y 7 con EE-SWAS con muestras homogéneas en sexo, etiología y tipo de trazado electroencefalográfico. La edad media de debut fue 3,6 años en grupo-SLK, debutando como regresión en lenguaje y conductual en 100% (5 pacientes inicialmente solo afectación expresiva); y 4 años en grupo-EE-SWAS, debutando como epilepsia en 100% y posterior regresión conductual. Un 57% de pacientes de grupo-SLK presentó epilepsia evolutiva con anomalías epilépticas en vigilia de predominio más posterior. La duración media de actividad epiléptica con punta-onda continua en sueño (SWAS) fue mayor en grupo-SLK (3,7 años) que grupo-EE-SWAS (1,8 años). Un 86% de los pacientes en ambos grupos mejoró con corticoides siendo la mejor opción terapéutica. A nivel neuropsicológico se constató deterioro cognitivo en el 71% del grupo-SLK y 43% grupo-EE-SWAS además de asociar dificultades atencionales en todos los pacientes EE-SWAS y en 85% de SLK.

**Conclusiones:**

EE-SWAS se caracteriza por regresión cognitivo-conductual, cuyo tratamiento son los corticoides. SLK es un subtipo de epilepsia dentro de este grupo con características propias como afectación inicial del lenguaje, actividad de electroencefalograma (EEG) posterior y mayor duración de SWAS.

## Abstract (en)

**Introduction:**

Landau-Kleffner Syndrome (LKS) is a subtype of epileptic encephalopathy with spike-wave activation during sleep (EESWAS), characterized by acquired aphasia associated with the emergence of epileptiform abnormalities.

**Patients and Methods:**

A cross-sectional descriptive study was conducted in a tertiary hospital, involving one group of children with LKS and another with EESWAS. The aim was to compare the clinical, neurophysiological, and neuropsychological aspects of both groups.

**Results:**

Seven patients with LKS and seven patients with EE-SWAS were analyzed; the samples were homogeneous in terms of sex, etiology, and type of electroencephalographic pattern. The mean ages of onset were 3.6 years in the LKS group, debuting with language and behavioral regression in 100% (five patients with expressive language impairment only), and 4 years in the EE-SWAS group, debuting with epilepsy in 100%, followed by behavioral regression. In 57% of the LKS group, evolving epilepsy was observed with predominantly posterior epileptic abnormalities during wakefulness. The mean duration of continuous spike-and-wave activity during sleep (SWAS) was longer in the LKS group (3.7 vs 1.8 years). Corticosteroids was the most effective treatment, with 86% of patients in both groups showing improvement. On a neuropsychological level, cognitive impairment was observed in 71% of the LKS group versus 43% in the EE-SWAS group; attention difficulties were present in all EE-SWAS patients and 85% of LKS patients.

**Conclusions:**

EE-SWAS is characterized by cognitive-behavioral regression, with corticosteroids being the treatment of choice. LKS is a subtype of epilepsy within this group, with distinct features such as initial language impairment, posterior electroencephalogram (EEG) activity, and a longer duration of SWAS.

## 1. Introducción

El Síndrome de Landau-Kleffner (SLK) es una encefalopatía epiléptica que se manifiesta en la infancia en niños con un desarrollo psicomotor previo normal. Su rasgo más distintivo es la afasia epiléptica adquirida caracterizada por una regresión progresiva del lenguaje y dificultades en el procesamiento auditivo. Es frecuente que asocien un deterioro cognitivo y trastornos del comportamiento [1]. Se presenta generalmente entre los 3 y 8 años con un pico de incidencia entre los 5 y los 7 años y tiene una proporción 2:1 de varones respecto a mujeres [2].

Es una enfermedad neurológica poco frecuente (0.2% de las epilepsias infantiles) [3]. En la clasificación de la Liga Interancional de la epilepsia (ILAE) de 2022 se clasifica como un subtipo específico dentro de las encefalopatías epilépticas con punta-onda continua durante el sueño (EE-SWAS, del inglés, Epileptic Encephalopathy Spike-Wave Activated Sleep) caracterizadas por una regresión cognitiva, lingüística, conductual y motora asociados temporalmente a un trazado de electroencefalograma caracterizado por la activación de punta-onda difusa durante todas las fases de sueño lento [3]. La exposición continua a la actividad epileptiforme en un cerebro inmaduro puede afectar la plasticidad necesaria para transferir el lenguaje a zonas homólogas contralaterales, lo que impacta en el desarrollo neurológico [4]. Las crisis clínicas se presentan en un 70% de los niños con SLK [5] y cuando se presentan, suelen ser de diferente semiología que incluyen: focales con conciencia alterada, clónicas y tónico-clónicas generalizadas y atónicas, generalmente con buen control farmacológico y la mayoría de los casos se resuelven al llegar a la adolescencia [2].

La nueva clasificación de la ILAE engloba el SLK dentro de las encefalopatías epilépticas tipo SWAS [3]. Aunque, clásicamente se ha considerado que los pacientes con SLK presentan características diferenciales respecto a la encefalopatía epiléptica con punta-onda continua activada en sueño lento (EE-SWAS), que han permitido considerarlo como una entidad en sí misma (Tabla 1, Ref. [1, 2, 3, 6]). En este sentido se describe que en los pacientes con SLK se produce inicialmente una pérdida de función limitada al lenguaje (afasia adquirida), mientras que en EE-SWAS el debut suele ser más precoz y se produce una afectación mayor de todas las funciones cognitivas [2, 7]. Asimismo, se han descrito diferencias en el patrón electro-encefalográfico: en vigilia en el SLK suelen aparecer puntas ondas en regiones centrotemporales bilaterales, que se describen como más limitadas a regiones posteriores (mientras que en EE-SWAS la distribución de las anomalías es fronto-central) el patrón punta-onda suele ocupar menor proporción del sueño non-rapid eye movement (NREM) que en EE-SWAS y, a diferencia de él, persiste durante el sueño rapid eye movement (REM) [3, 6]. Además, las alteraciones estructurales objetivadas en la Resonancia Magnética (RM) cerebral son más frecuentes en la EE-SWAS (polimicrogiria, lesiones talámicas…) que en el SLK [1, 2].

**Tabla 1.** **Comparación de las características electro-clínicas entre EE-SWAS y SLK descritas clásicamente en la bibliografía [1, 2, 3, 6]**.

EE-SWAS	SLK
Incidencia 0,6–0,7%	Incidencia 0,2%
Debut 2–12 años, pico 4–5 años	Debut 3–8 años, pico 5–7 años
Etiología: estructural (polimicrogiria, lesiones talámicas), genética (*GRIN2A*), metabólica, idiopática	Etiología: desconocida
	Reciente relación causa genética (*GRIN2A*)
Afectación en todas las funciones cognitivas y conductuales	Afectación lenguaje (afasia adquirida)
Debut con crisis 80%, crisis evolutivas casi 100%	Debut con crisis 60%, crisis evolutivas 70%
Patrón EEG: punta-onda más proporción del sueño NREM y menos en REM	Patrón EEG: punta-onda menos proporción del sueño NREM y más en REM
Actividad epileptiforme tipo punta onda lenta en regiones frontro centrales/temporales	Actividad epileptiforme tipo punta onda en regiones centrotemporales y posteriores (temporales posteriores y parieto occipitales)

EE-SWAS, encefalopatía epiléptica con punta-onda activada durante el sueño; SLK, Síndrome de Landau-Kleffner; EEG, electroencefalograma; NREM, non-rapid eye movement; REM, rapid eye movement. Estas diferencias entre ambos grupos se basan en artículos en los que se describen series de escasos pacientes y de características heterogéneas. El objetivo de este trabajo es analizar las diferencias entre grupos homogéneos de pacientes con SLK y EE-SWAS y comparar con lo descrito clásicamente.

## 2. Material y Métodos

Se ha realizado un estudio transversal y descriptivo con muestreo consecutivo en el servicio de Neuropediatría del Hospital Niño Jesús de Madrid. Se incluyeron únicamente pacientes con etiología desconocida y registros completos para minimizar sesgos de selección. Se incluyeron 7 pacientes con edades comprendidas entre los 0 y los 18 años que habían sido diagnosticados de SLK de etiología no conocida en los que se hubiera realizado al menos un video-EEG que incluyera sueño y una valoración neuropsicológica completa. Además, se incluyó un grupo control de 7 pacientes diagnosticados de EE-SWAS sin etiología identificada. Ambos grupos eran homogéneos respecto edad, sexo, etiología idiopática y desarrollo psicomotor previo normal.

Para ello se realizó una revisión a través de historias clínicas de pacientes con SLK y EE-SWAS y se recogieron variables clínicas, demográficas (edad, sexo), antecedentes perinatales, edad de inicio de aparición de la encefalopatía epiléptica, crisis, tipo de epilepsia y evolución. Los tipos de crisis y las características eléctricas (características del trazado y trazado SWAS) se clasificaron utilizando la clasificación ILAE de 2022 [3]. En todos los pacientes se había realizado un video-EEG que incluyera sueño y se analizaron las características del trazado y la presencia de punta-onda continua en sueño (SWAS). Se analizaron los tratamientos empleados (fármacos anti-crisis, dieta cetogénica, inmunoterapia o técnicas quirúrgicas). La eficacia en los tratamientos empleados se definió desde el punto de vista clínico en forma de mejoría o control total de crisis y desde el punto de vista eléctrico con mejoría de las anomalías y desaparición de trazado SWAS en el EEG a los 3 meses de iniciado el tratamiento. Se recogieron las variables de los diferentes test neuropsicológicos realizados (Tabla 2).

**Tabla 2.** **Batería de pruebas neuropsicológicas empleadas**.

Test Neuropsicológico	Función Cognitiva
WISC-V	Test de inteligencia
Memoria Verbal de RIAS-2	Test de inteligencia
Memoria No Verbal RIAS-2	
Batería NEPSY-II	Atención y función ejecutiva, Lenguaje, Memoria y aprendizaje, Percepción social, Procesamiento visoespacial y Sensoriomotor.
K-ABC-2	Procesamiento mental y capacidad cognitiva
Pegboard WRAVMA	Test de manipulación y destreza
BRIEF 2	Evaluación conductual de la función ejecutiva
SENA	Conducta y problemas emocionales
Escalas VABS-III, escala Vineland	Habilidades conceptuales, sociales y prácticas aprendidas

WISC-V, Test de inteligencia de Weschler para niños-V; RIAS-2, Reynolds Intellectual Assessment Scales, Second Edition; NEPSY-II, A Developmental Neuropsychological Assessment, Second Edition; K-ABC-2, Kaufman Assessment Battery for Children; WRAVMA, Wide Range Assessment of Visual Motor Ability (Cuestionario de Lateralidad Manual de Oldfield); BRIEF 2, Behavior Rating Inventory of Executive Function, Second Edition; SENA, Sistema de Evaluación de Niños y Adolescentes; VABS-III, Escalas Vineland de Conducta Adaptativa, Tercera Edición.

Las variables cuantitativas se expresaron en forma de media y desviación típica. Las variables cualitativas se expresaron como frecuencias absolutas y porcentajes. Los análisis estadísticos se realizaron con el programa SPSS versión 22.0 (IBM Corp., Armonk, NY, USA). Se empleó una prueba estadística paramétrica basada en la distribución normal (prueba Z para la diferencia de proporciones), con un nivel de significación estadística establecido en *p* < 0,05.

## 3. Resultados

**Características de la muestra y etiología**: Se incluyeron un total de 7 pacientes con SLK según criterios clínicos y eléctricos, de los cuales 2 eran varones y 5 mujeres. En el grupo comparativo con encefalopatía epiléptica con SWAS, también se analizaron 7 pacientes, con 4 varones y 3 mujeres. Ninguno de los dos grupos presentaba antecedentes perinatales relevantes, y todos los pacientes mostraban un desarrollo psicomotor normal antes del inicio de la epilepsia. En cuanto a la etiología, no se encontraron lesiones causales en la RM. Como hallazgos incidentales en el grupo SLK, se identificó un paciente con quiste de la bolsa de Rathke, un paciente quiste aracnoideo y pequeñas hiperintensidades periatriales inespecíficas en otro, no causales con respecto a su epilepsia. Desde el punto de vista genético, no se detectaron mutaciones en el exoma clínico de los pacientes estudiados, realizándose análisis en 6 de 7 pacientes del grupo SLK y 3 de 7 del grupo EE-SWAS.

**Características de la Epilepsia**: En el grupo-SLK, la edad media de debut clínico fue de 3,6 años (DT 1,2), donde el 100% de los pacientes presentaron regresión en el lenguaje como síntoma inicial. En 5 de los 7 pacientes (71%), la afectación inicial fue únicamente en el lenguaje expresivo, con posterior desarrollo en todos ellos de agnosia auditiva y dificultades en la comprensión del lenguaje. Además, todos los pacientes mostraron síntomas de alteración conductual junto con la regresión del lenguaje. Solo el 57% de los pacientes presentó epilepsia evolutiva con crisis focales motoras.

En el grupo EE-SWAS, la edad media de debut fue de 4 años (DT 1,3), comenzando en el 100% de los casos con la epilepsia, presentando esta una considerable mayor probabilidad en este grupo (100%) que en SLK (57%) (*p* < 0,05), seguida de una regresión conductual. Cuatro de los siete pacientes presentaban previamente una epilepsia autolimitada con puntas-ondas centrotemporales, evolucionando posteriormente a EE-SWAS, mientras que tres debutaron directamente con esta encefalopatía. Respecto al tipo de crisis en este grupo, se observaron crisis focales motoras en 7 de 7 pacientes, 1 con crisis mioclónicas, 2 con crisis de ausencia y 2 con mioclono-negativo.

Electroencefalográficamente, todos los pacientes presentaron punta-onda generalizada difusa en sueño NREM, con anomalías epilépticas centro-temporales predominantes. En el grupo SLK, 5 de 7 pacientes mostraron anomalías tipo punta-onda en regiones centrotemporales y 2 en regiones parieto-occipitales. En el grupo EE-SWAS, 6 de 7 presentaron patrones de punta-onda en regiones centrotemporales, no presentando una diferencia estadísticamente significativa respecto al grupo SLK (*p* = 0,51) y 1 en región frontal.

**Fig. 1.**
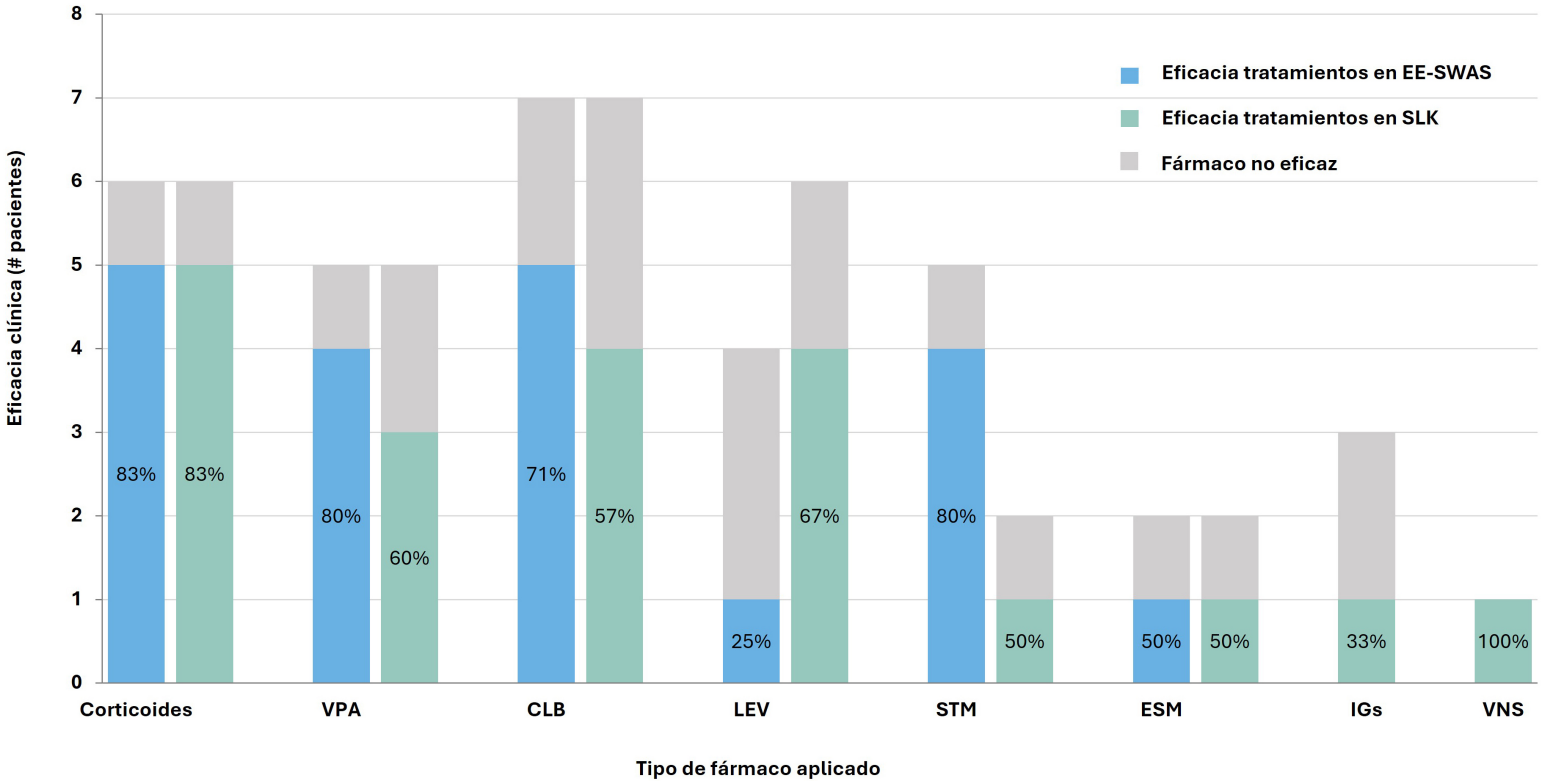
**Eficacia de los tratamientos empleado en ambos grupos (EE-SWAS, SLK)**. VPA, ácido valproico; CLB, clobazam; LEV, levetiracetam; STM, sultiame; ESM, etosuximida; IGs, inmunoglobulinas; VNS, estimulador del nervio vago.

En cuanto al tratamiento, la media de fármacos anticrisis (FACs) fue de 5,1 (DT 3,4) en el grupo SLK y 3,7 (DT 0,8) en el grupo EE-SWAS. Los fármacos más frecuentemente utilizados fueron ácido valproico, clobazam, levetiracetam, sultiame, etosuximida y corticoides (Fig. 1). Respecto a la eficacia, los corticoides mostraron buena respuesta en ambos grupos (eficacia 86% con este tratamiento). Los FAC con mayor respuesta fueron el ácido valproico, clobazam, levetiracetam y el sultiame. Los fármacos con menor eficacia fueron la carbamazepina y el brivaracetam.

Las pautas de corticoides en el grupo SLK fueron heterogéneas debido a que en muchas ocasiones se habían iniciado en otros centros hospitalarios e incluyeron tratamientos como hidrocortisona (5 mg/kg/día), prednisona oral (1 mg/kg/día), ACTH (1 mes) + hidrocortisona (2 mg/kg/día), y dexametasona (0,6 mg/kg/día). En contraste, en el grupo EE-SWAS se utilizó un régimen estandarizado, de ciclos mensuales de metilprednisolona (30 mg/kg/día IV durante 3 días) y prednisona oral (1–2 mg/kg/día durante 4 días), manteniendo las pautas por al menos 3 meses. Hay que tener presente que esta variabilidad terapéutica puede constituir un factor que dificulte la comparación de la eficacia entre grupos.

La recurrencia de trazado SWAS tras la respuesta al tratamiento con corticoides fue del 50% en el grupo SLK y del 33% en el grupo EE-SWAS. La duración media del trazado SWAS fue mayor en SLK (3,7 años, DT 3) que en EE-SWAS (1,8 años, DT 0,9).

**Características Neuropsicológicas**: A nivel neuropsicológico, se evidenció un deterioro cognitivo en el 71% de los pacientes del grupo SLK y en el 43% del grupo EE-SWAS, no presentando una diferencia estadísticamente significativa (*p* = 0,29), en comparación de las evaluaciones neuropsicológicas antes y después del inicio trazado SWAS (Tabla 3). Cabe destacar que las evaluaciones neuropsicológicas se realizaron en distintos momentos del proceso clínico para cada paciente, sin un protocolo temporal uniforme, lo cual podría afectar la comparabilidad de los resultados. Ambos grupos desarrollaron alteraciones conductuales, especialmente trastornos del control de impulsos y trastornos disejecutivos, observándose en el 100% de los pacientes de EE-SWAS y en el 85% de los del SLK. Además, síntomas de inquietud motriz se manifestaron en la mitad de los pacientes de ambos grupos. Un paciente de cada grupo desarrolló síntomas de trastorno del espectro autista, mientras que un paciente del grupo SLK presentó un trastorno psicótico.

**Tabla 3.** **Comparación de las características neuropsicológicas entre los grupos SLK y EE-SWAS**.

Características Neuropsicológicas	EE-SWAS	SLK	Significación estadística (*p*-valor)
Deterioro cognitivo	Deterioro cognitivo global: 43%	Deterioro cognitivo global: 71%	*p* = 0,29
	-CIT límite 14%	-CIT límite 28%	
	-CIT DI leve 28%	-CIT DI leve 14%	
		-CIT DI moderada 28%	
Dificultades atencionales (TDA)	100%	85%	*p* = 0,29
Trastornos conductuales	100%	85%	*p* = 0,29
Inquietud motriz	57%	57%	*p* = 1,00
Rasgos TEA	14%	14%	*p* = 1,00
Trastorno psicótico	0%	14%	*p* = 0,30

CIT, coeficiente intelectual; DI, discapacidad intelectual; TDA, trastorno por déficit de atención; TEA, trastorno del espectro autista.

## 4. Discusión

SLK y EE-SWAS son epilepsias poco frecuentes en la población pediátrica, lo que limita el conocimiento sobre cómo sus características electro-clínicas influyen en las alteraciones neurocognitivas. En este estudio se comparan estas dos condiciones en términos de características clínicas, eléctricas, tratamientos y aspectos neurocognitivos.

Desde el punto de vista clínico, nuestros hallazgos concuerdan con la literatura existente. En el grupo de EE-SWAS, todos los pacientes presentaron debut con epilepsia, seguido de una regresión cognitiva predominantemente conductual. En contraste, el grupo SLK inició con alteraciones del lenguaje como primer signo clínico, observándose que solo el 57% de los pacientes del SLK experimentó crisis epilépticas, mientras que todos los pacientes en el grupo EE-SWAS lo hicieron (*p* < 0,05).

Tradicionalmente, se ha considerado que la agnosia auditiva verbal es un síntoma característico del SLK, comenzando con la incapacidad para comprender el lenguaje oral y avanzando a dificultades en la expresión verbal [2, 7]. Sin embargo, en nuestra serie, el primer signo clínico observado fue la afectación del lenguaje expresivo con afasia adquirida, aunque evolutivamente también se evidenció la afectación en la comprensión. Tener presente este signo precoz como patrón de SLK permitiría un diagnóstico y tratamiento más precoz en esta encefalopatía epiléptica. Las alteraciones conductuales fueron comunes en ambos grupos, lo que difiere de estudios previos [1, 3, 7] que consideraban este aspecto más propio de la EE-SWAS.

En cuanto a los hallazgos electroencefalográficos, nuestro estudio se alinea con la serie de Caraballo *et al*. [2], y con otro artículo de Stefanatos [6], que reportaron anomalías en las regiones centrotemporales y temporo-occipitales en pacientes con SLK. En nuestra muestra, las anomalías EEG predominantes fueron centrotemporales en ambos grupos, aunque se observaron anomalías frontales en EE-SWAS y temporo-occipitales en SLK. El trazado de punta-onda continua en sueño se registró principalmente en sueño NREM en ambos grupos de pacientes sin distinguir una mayor proporción en sueño REM en pacientes con SLK como se describe previamente en la literatura [3, 6].

Curiosamente, la duración del trazado con punta-onda continua en sueño fue mayor en los pacientes con SLK (3,7 años, DT 3) que en los de EE-SWAS (1,8 años, DT 0,9), a diferencia de lo reportado en la literatura donde el trazado SWAS en el SLK suele tener menor duración [2]. Esto podría explicarse porque en el SLK, el inicio de los síntomas no está asociado a las crisis epilépticas, lo que podría retrasar el diagnóstico y tratamiento.

Respecto al tratamiento, los corticoides se identificaron como la opción más eficaz en ambos grupos, corroborando hallazgos de otros estudios [8, 9, 10, 11]. En la serie de Buzatu* et al*. [12], se describen 44 pacientes con EE-SWAS con mejoría en 21 pacientes, y una recurrencia de un 44%, similar al porcentaje descrito en nuestro estudio; también en este el uso de otras medicaciones anticrisis mostró similitudes entre los grupos, mostrando que tanto el clobazam (eficacia alrededor de 65%) y el ácido valproico (eficacia alrededor 70%) fueron eficaces. En nuestra serie el Levetiracetam parece ser más efectivo en el grupo-SLK (67 % versus 25%) mientras que el sultiame fue más eficaz en el grupo-EE-SWAS. Esto podría explicarse porque en 4 pacientes del grupo EE-SWAS habían evolucionado desde una epilepsia autolimitada con puntas-ondas centrotemporales, donde se ha demostrado que el sultiame es una opción eficaz [13, 14].

El conocimiento sobre el funcionamiento neurocognitivo y comportamental en pacientes con SLK y EE-SWAS es limitado. En nuestra serie, se registró un deterioro cognitivo en ambos grupos, siendo más acentuado en el SLK con diferencias más significativas en el índice de comprensión verbal, posiblemente secundario a una mayor duración del trazado con punta-onda continua. Hay que tener en cuenta que el deterioro cognitivo puede estar influido por múltiples factores (edad, tipo de crisis, duración del trazado SWAS, tratamiento, entre otros), lo que dificulta su control en nuestra pequeña muestra. Las dificultades atencionales fueron las alteraciones neurocognitivas más frecuentes manifestándose como déficit de atención, inquietud motriz e impulsividad. Las alteraciones conductuales afectaron aproximadamente a la mitad de los pacientes de ambos grupos. Estos hallazgos son consistentes con la literatura, que reporta comorbilidades conductuales en estas encefalopatías.

Consideramos fundamental que el entendimiento de las características cognitivas y conductuales de estos pacientes podría mejorar el manejo clínico y orientar tratamientos más específicos, dado que actualmente no existen intervenciones cognitivo-conductuales dirigidas a estas condiciones.

El pronóstico de la epilepsia es generalmente favorable, con resolución de crisis y anomalías EEG durante la adolescencia y buena respuesta a medicación. Sin embargo, el pronóstico cognitivo y conductual es peor, con afectación variable del lenguaje en los niños con SLK [1]. En nuestra serie, observamos mayor deterioro cognitivo en el SLK que en la literatura [2], posiblemente relacionado con la duración del trazado SWAS. Respecto a la etiología, aunque las resonancias magnéticas mejoradas permiten identificar anomalías estructurales en el neurodesarrollo, no se encontraron en nuestros pacientes con SLK. La relación entre mutaciones genéticas y SLK/EE-SWAS está siendo estudiada, y recientemente se han propuesto nuevos genes candidatos implicados en el desarrollo de estas encefalopatías [15, 16], siendo el gen *GRIN2A*, relacionado con el receptor NMDA implicado en el desarrollo cerebral y la memoria [17], el que más se asocia en la actualidad. Las mutaciones en el gen *GRIN2A *pueden alterar la capacidad del receptor para responder adecuadamente al glutamato, provocando un desajuste en la excitación neuronal y con ello crisis epilépticas, además se han asociado con diversas presentaciones clínicas, desde trastornos más leves a más graves con afectación cognitiva. En nuestra serie no se observaron mutaciones causales en los análisis genéticos realizados lo que puede deberse al tamaño muestral o a limitaciones técnicas en el diagnóstico.

## 5. Limitaciones

Este estudio presenta algunas limitaciones que deben ser consideradas al interpretar los resultados. En primer lugar, el tamaño muestral reducido (n = 14 en total) puede limitar la generalización de los resultados obtenidos, además el diseño transversal descriptivo conlleva limitaciones inherentes, como la posibilidad de sesgos en la recolección de datos debido a la falta de información estandarizada y la variabilidad en el registro clínico. Además, el uso de datos retrospectivos extraídos de historias clínicas puede introducir sesgos relacionados con la calidad, completitud y cronología de la información disponible.

Por otra parte, hay que tener presente que la variabilidad terapéutica, especialmente en el grupo SLK, puede representar una posible fuente de confusión al comparar la eficacia entre grupos En cuanto a las evaluaciones cognitivas, estas se basaron en pruebas realizadas previamente, lo que implica posibles diferencias en los instrumentos utilizados y en los intervalos de tiempo entre evaluaciones. La ausencia de reevaluaciones a largo plazo limita, asimismo, la posibilidad de analizar con mayor profundidad la evolución del deterioro cognitivo en esta población.

## 6. Conclusiones

Una regresión en el lenguaje debe considerarse un signo de alarma para el inicio de una encefalopatía epiléptica, particularmente en el SLK, que se presenta como un subtipo dentro del espectro de EE-SWAS con unas características específicas como la afectación temprana del lenguaje en forma de afasia, unas anomalías electro-encefalográficas más posteriores y una mayor duración de la SWAS; con características comunes como la regresión cognitiva y afectación conductual. En este contexto, en nuestra serie limitada de pacientes, los corticoides fue el tratamiento que mostró mayor eficacia clínica y eléctrica tanto en EE-SWAS como en SLK, lo que subraya la importancia de iniciar su administración de manera temprana para optimizar los resultados clínicos.

## Data Availability

Todos los datos generados o analizados durante este estudio están incluidos en este artículo. No se requieren datos adicionales para reproducir los resultados.

## Contribuciones de los Autores

CBP diseñó el estudio, revisó los registros de los pacientes, realizó el análisis estadístico y redactó el manuscrito. VSI y NLC contribuyeron al diseño del estudio y a la redacción del manuscrito. EGA, JJGP y ADR colaboraron en la concepción o diseño del trabajo, contribuyeron a la redacción de los resultados y conclusiones, y proporcionaron una revisión crítica del manuscrito. MGF contribuyó a la descripción e interpretación de los EEG, además de revisar críticamente el manuscrito. BEO realizó las evaluaciones neuropsicológicas y contribuyó a la interpretación de esos resultados. Todos los autores contribuyeron a los cambios editoriales del manuscrito. Todos los autores leyeron y aprobaron el manuscrito final. Todos los autores han participado lo suficiente en el trabajo y han acordado ser responsables de todos los aspectos del mismo.

## Aprobación Ética y Consentimiento Informado

El proyecto de investigación fue aprobado por el Comité de Ética de la Investigación con Medicamentos del Hospital Universitario Niño Jesús (CEIM), código interno: R-0027/25. Todos los pacientes firmaron un consentimiento informado, de acuerdo con la Declaración de Helsinki.

## Agradecimientos

No aplicable.

## Financiación

Esta investigación no recibió financiación externa.
